# Machine learning-based prediction of cerebral hemorrhage in patients with hemodialysis: A multicenter, retrospective study

**DOI:** 10.3389/fneur.2023.1139096

**Published:** 2023-04-03

**Authors:** Fengda Li, Anmin Chen, Zeyi Li, Longyuan Gu, Qiyang Pan, Pan Wang, Yuechao Fan, Jinhong Feng

**Affiliations:** ^1^Department of Neurosurgery, Changshu Hospital Affiliated to Soochow University, Changshu, China; ^2^Department of Nephrology, The First People's Hospital of Jintan, Changzhou, China; ^3^School of Computer Science, Nanjing University of Posts and Telecommunications, Nanjing, China; ^4^Department of Neurosurgery, Affiliated Hospital of Xuzhou Medical University, Xuzhou, China; ^5^Faculty of Informatics, Università della Svizzera italiana, Lugano, Ticino, Switzerland; ^6^Department of Nephrology, Affiliated Hospital of Xuzhou Medical University, Xuzhou, China

**Keywords:** hemodialysis, uremia, intracerebral hemorrhage, machine learning, predictive models, Shapley additive explanations

## Abstract

**Background:**

Intracerebral hemorrhage (ICH) is one of the most serious complications in patients with chronic kidney disease undergoing long-term hemodialysis. It has high mortality and disability rates and imposes a serious economic burden on the patient's family and society. An early prediction of ICH is essential for timely intervention and improving prognosis. This study aims to build an interpretable machine learning-based model to predict the risk of ICH in patients undergoing hemodialysis.

**Methods:**

The clinical data of 393 patients with end-stage kidney disease undergoing hemodialysis at three different centers between August 2014 and August 2022 were retrospectively analyzed. A total of 70% of the samples were randomly selected as the training set, and the remaining 30% were used as the validation set. Five machine learning (ML) algorithms, namely, support vector machine (SVM), extreme gradient boosting (XGB), complement Naïve Bayes (CNB), K-nearest neighbor (KNN), and logistic regression (LR), were used to develop a model to predict the risk of ICH in patients with uremia undergoing long-term hemodialysis. In addition, the area under the curve (AUC) values were evaluated to compare the performance of each algorithmic model. Global and individual interpretive analyses of the model were performed using importance ranking and Shapley additive explanations (SHAP) in the training set.

**Results:**

A total of 73 patients undergoing hemodialysis developed spontaneous ICH among the 393 patients included in the study. The AUC of SVM, CNB, KNN, LR, and XGB models in the validation dataset were 0.725 (95% CI: 0.610 ~ 0.841), 0.797 (95% CI: 0.690 ~ 0.905), 0.675 (95% CI: 0.560 ~ 0.789), 0.922 (95% CI: 0.862 ~ 0.981), and 0.979 (95% CI: 0.953 ~ 1.000), respectively. Therefore, the XGBoost model had the best performance among the five algorithms. SHAP analysis revealed that the levels of LDL, HDL, CRP, and HGB and pre-hemodialysis blood pressure were the most important factors.

**Conclusion:**

The XGB model developed in this study can efficiently predict the risk of a cerebral hemorrhage in patients with uremia undergoing long-term hemodialysis and can help clinicians to make more individualized and rational clinical decisions. ICH events in patients undergoing maintenance hemodialysis (MHD) are associated with serum LDL, HDL, CRP, HGB, and pre-hemodialysis SBP levels.

## 1. Introduction

Maintenance hemodialysis (MHD) is the primary renal replacement therapy for patients with uremia (1). Intracerebral hemorrhage (ICH), defined as non-traumatic hemorrhage in the brain parenchyma with or without ventricles, accounts for 10–15% of all stroke cases and is an important cause of disability and death globally (2). ICH is one of the most serious complications among patients undergoing MHD. Various factors have an important impact on the occurrence and development of ICH. Recent studies have attempted to identify relevant risk factors, and lipid metabolism and inflammatory responses have been reported as important factors regulating the progression of ICH and subsequent brain injury and brain function repair. Despite the continuous development of hemodialysis technology and the gradual improvement of nursing levels, the risk of a cerebral hemorrhage in patients undergoing MHD is approximately six times higher than that in healthy individuals (3), and the mortality rate is as high as 41–47% (4). Most patients require admission to the intensive care unit (ICU) for monitoring and treatment, which imposes a serious economic burden on the family and society.

ICH often has no identifiable warning signs or symptoms. Although optimal strategies for the medical and surgical management of ICH have been investigated, survival and functional outcomes have not been significantly improved (5). Therefore, establishing risk prediction models to identify high-risk patients undergoing MHD is important for the early implementation of targeted interventions. To date, only a few studies have attempted to develop such models.

Machine learning (ML), an artificial intelligence method, uses computers to statistically learn from datasets and build corresponding models to identify relationships between various factors. In the field of medicine, ML is increasingly used through statistical learning methods to overcome possible obstacles in clinical practice (6, 7). In recent years, although ML has been used to analyze clinical data to predict the complications and adverse outcomes of critical illnesses (8–10), few efforts have been made to develop strategies for predicting the prognosis of patients with uremia undergoing dialysis, especially for predicting the risk of cerebral hemorrhage, a serious complication of dialysis. ML has shown good performance in previous studies; however, because of its “black box” nature, the effects of each feature on the final results remain unknown, and it is difficult to explain the factors that lead to a given prediction. This lack of interpretability limits the widespread application of ML methods in medical research (11, 12). Shapley additive explanation (SHAP) is a method inspired by the classical game theory that assigns a predicted value to each feature and evaluates the contribution of each feature to the results of ML models to achieve a balance between the accuracy and interpretability of the model (13).

To analyze complex variables that may be related to a cerebral hemorrhage after regular hemodialysis, we integrated the demographic data, laboratory test results, hemodialysis indicators, and other information of patients to construct a model for predicting the risk of a cerebral hemorrhage. To make the model more applicable for the diagnosis of chronic kidney disease with intracerebral hemorrhage, overcome the “black box” nature of ML, and explore the relationship between each feature and its clinical significance, we used the extreme gradient boosting (XGBoost) algorithm to develop the model (14). SHAP was used to provide a more intuitive global and local explanation of the model to understand the prediction of the model and improve the clinical understanding of the risk of a cerebral hemorrhage in patients with hemodialysis.

## 2. Materials and methods

### 2.1. Study population and data source

Patients with end-stage kidney disease undergoing hemodialysis from August 2014 to August 2022 at the Affiliated Hospital of Xuzhou Medical University, Xuzhou Central Hospital, and the Second Affiliated Hospital of Xuzhou Medical University were recruited for the study. According to the occurrence of ICH, the patients were divided into ICH and non-ICH groups.

### 2.2. Data collection

The inclusion criteria were as follows: (a) patients diagnosed with uremia according to chronic kidney disease (CKD) staging and recommendations or the Kidney Disease Outcomes Quality Initiative (KDOQI) guidelines formulated by the American Kidney Foundation, that is, patients with estimated glomerular filtration rate (eGFR) of < 15 ml/(min·1.73 m^2^) diagnosed with CKD stage 5, which is the uremia stage (15); (b) patients receiving hemodialysis regularly, those aged ≥18 years, those with dialysis age of ≥3 months, and dialysis frequency of three times per week and 4 h per dialysis; and (c) patients with ICH confirmed *via* a CT examination of the head. The exclusion criteria were as follows: (a) patients with severe failure of the heart, lung, and other organs, blood system diseases, autoimmune diseases, and malignant tumors; (b) patients with primary subarachnoid hemorrhage, secondary cerebral hemorrhage, such as trauma, intracranial tumors, ICH caused by hemorrhage after an ischemic stroke, and severe coagulation dysfunction; (c) patients on antiplatelet drugs, hormones, immunosuppressants, and antibacterial agents in the past 1 month; and (d) patients with missing clinical data. Based on the diagnosis and inclusion and exclusion criteria, 393 patients with end-stage kidney disease complicated with cerebral hemorrhage owing to long-term hemodialysis were included. Of these 393 patients, 73 patients were included in the ICH group, whereas 320 patients were included in the non-ICH group. Because this study had a retrospective design, there was no security-related risk. The present study was approved by the Ethics Committee of the Affiliated Hospital of Xuzhou Medical University.

### 2.3. Inclusion of observed variables

The clinical data of patients were collected with reference to clinical experience, reported literature, and medical records in the electronic medical record systems of the three centers. Data regarding the following five aspects were collected: (1) demographic data (sex and age); (2) vascular risk factors (hypertension, diabetes, polycystic kidney disease, and duration of dialysis); (3) baseline blood pressure (systolic blood pressure [SBP] and diastolic blood pressure [DBP] before and after dialysis); (4) treatment during hemodialysis (including anticoagulant dosage, dialysis access, and blood flow velocity); and (5) laboratory tests (white blood cells [WBCs], platelets [PLTs], hemoglobin [HGB], neutrophils [Nes], lymphocytes [Lys], hematocrit [HCT], C-reactive protein [CRP], neutrophil-to-lymphocyte ratio [NLR], platelet-to-lymphocyte ratio [PLR], alanine aminotransferase [ALT], aspartate aminotransferase [AST], serum total protein [TP], serum albumin [ALB], blood urea nitrogen [BUN], serum creatinine [Scr], cystatin C [CysC], eGFR, uric acid [UA], triglyceride [TG], total cholesterol [TC], low-density lipoprotein [LDL], high-density lipoprotein [HDL], blood potassium [K], blood sodium [Na], blood calcium [Ca], calcium–phosphorus product, and blood phosphorus [P]).

### 2.4. Selection of machine learning models

Before constructing ML models, the original clinical data were normalized. Normalization can improve the speed of gradient descent to find the optimal solution, and the algorithm for Euclidean distance can effectively improve the accuracy. In this study, the min–max normalization method was used to normalize the characteristic values of clinical data to the range of (0,1).

Approximately 70% of the samples in the dataset were randomly selected as the training set, whereas the remaining 30% of the samples were used as the validation set. The dataset is represented as *D* = {(*x*_*i*_, *y*_*i*_), *i* = 1, 2, …, *N*}, where *x*_*i*_ *is* [*x*_*i*1_, *x*_*i*2_, *x*_*i*3_, …, *x*_*ip*_], which is a row vector with input variables (or features) of real value as its elements, and *y*_*i*_∈{0, 1} is a scalar with the output of an integer value as its element. The task in hand was a binary classification problem, that is, the generation of a model (*y* = *f*[*x*]) in the training set. The model was subsequently verified in the validation set to predict $\hat{y_{k}}=f\left(x_{k}\right)$. The predicted output $\hat{y_{k}}\;$should be similar to the actual output. All models were tested using Python.

We applied five ML algorithms to model the data: logistic regression (LR), support vector machine (SVM), K-nearest neighbor (KNN), complement Naive Bayes (CNB), and XGBoost. To be able to ensure that the training samples selected for multiple-model training were consistent, we generalized the performance of each model over multiple training sessions using a resampling training/validation mechanism. The XGBoost (version 1.2.1), lightGBM (version 3.2.1), and sklearn (version 0.22.1) packages were used for developing the ML models. For the RF algorithm, “ntree” was set to 100, and “mtree” was set to 3. To avoid overfitting and enhance interpretability, the maximum tree depth was set to eight nodes in the XGBoost algorithm. In addition, to evaluate the predictive accuracy of various ML models, accuracy, precision, sensitivity, specificity, F1 score, and the area under the receiver operating characteristic curve (ROC) were evaluated.

SHAP is a “model interpretation” package developed based on Python. To understand the results of the model output, the SHAP package was used to interpret and sort the features of the trained model and examine the contribution of each element in the features to the model.

### 2.5. Statistical analysis

The R software (version 4.02) was used for data processing and statistical analysis. Categorical variables were expressed in terms of quantity and percentage and were compared using Fisher's exact test or the chi-square test. For continuous variables, the Shapiro–Wilk test was initially used to determine whether the variables conformed to a normal distribution, and the independent sample *t*-test (conforming to a normal distribution) was subsequently used for comparing the data, which were expressed as mean ± standard deviation. The Mann–Whitney *U*-test was used to compare data with non-normal distribution, which were expressed as the median (first and third quartiles). A *P* < 0.05 was considered statistically significant.

## 3. Results

### 3.1. Baseline patient characteristics

**Figure 1:**
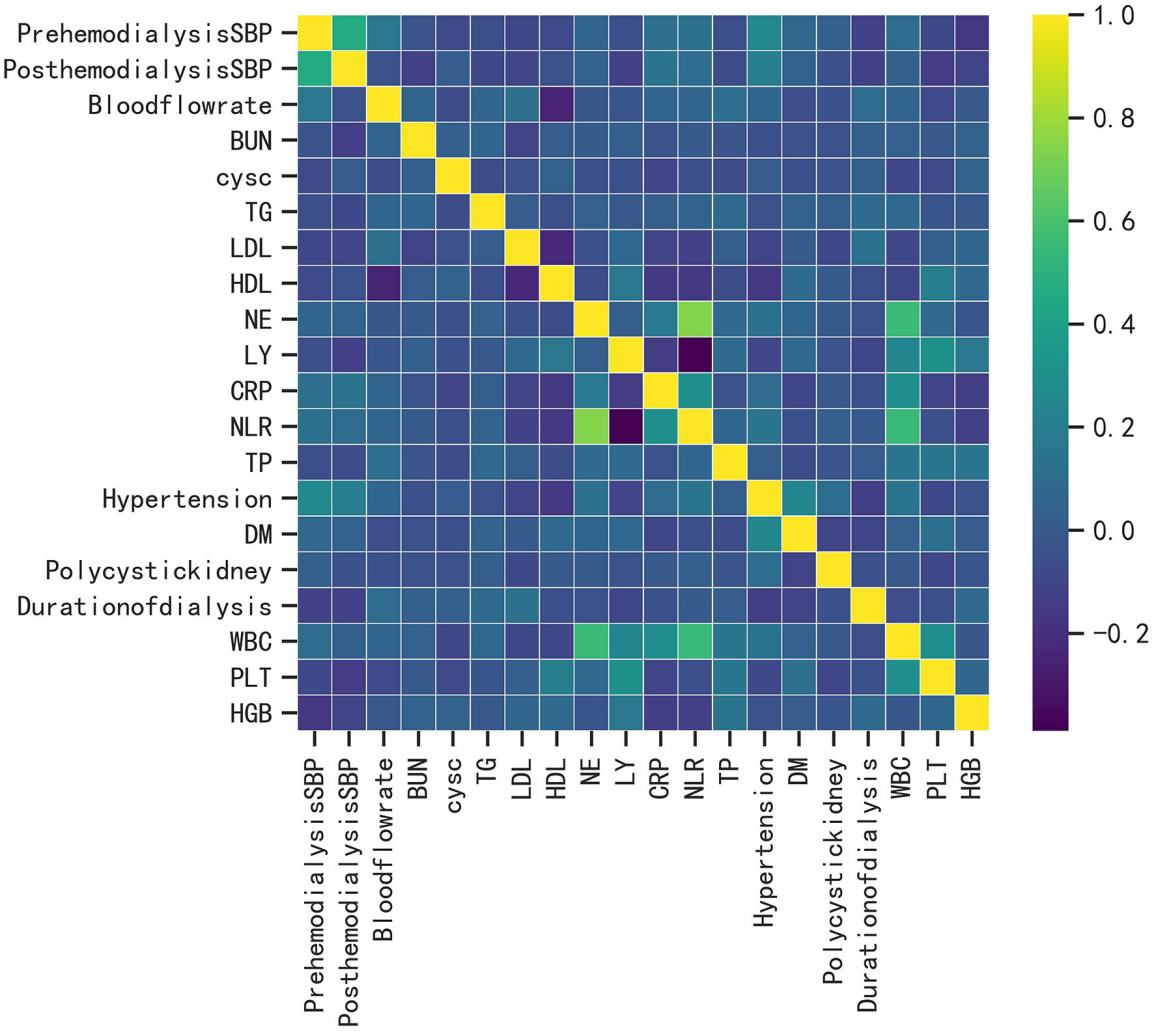
Heat map of the correlation of patient's clinical features.

A total of 393 patients were included in this study, and the baseline characteristics of the ICH and non-ICH groups are shown in Table 1. In terms of demographic characteristics, no significant differences were observed in the sex and age of patients between the two groups. The history of diabetes and polycystic kidney disease was a significant variable in terms of underlying diseases. The blood flow rate and SBP before and after dialysis were important variables in terms of dialysis indicators. Laboratory indices, such as the levels of CRP, LDL, and HDL, were significantly different between the two groups. We further constructed a heat map demonstrating Spearman correlation coefficients to visualize the correlation between variables with differences (Figure 1).

**Table 1:** Baseline features of patients.

**Variables**	**Non-ICH (*n* = 320)**	**ICH (*n* = 73)**	***P*-value**
Age (years)	57.000 (46.000, 66.000)	54.000 (48.000, 63.000)	0.384
Sex (%)			0.697
Female	108 (33.7)	27 (37.0)	
Male	212 (66.3)	46 (63.0)	
Hypertension (%)			< 0.001
No	141 (44.1)	13 (17.8)	
Yes	179 (55.9)	60 (82.2)	
Diabetes mellitus (%)			0.001
No	196 (61.2)	60 (82.2)	
Yes	124 (38.8)	13 (17.8)	
Polycystic kidney (%)			0.001
No	311 (97.2)	64 (87.7)	
Yes	9 (2.8)	9 (12.3)	
Duration of dialysis (months)	41.84 (19.27, 67.99)	34.17 (18.67, 43.97)	0.014
WBCs (10^9^/L)	5.60 (4.58, 6.82)	6.60 (4.91, 8.85)	< 0.001
PLTs (10^9^/L)	165.00 (131.75, 204.25)	145.00 (117.00, 180.00)	0.009
HGB (g/L)	106.55 (16.77)	92.43 (13.26)	< 0.001
NE (10^9^/L)	3.82 (3.02, 4.91)	5.16 (3.51, 7.47)	< 0.001
LY (10^9^/L)	1.00 (0.80, 1.30)	0.82 (0.60, 1.10)	< 0.001
HCT (%)	33.27 (5.71)	31.87 (6.07)	0.061
CRP (mg/L)	5.16 (3.16, 7.20)	12.00 (4.79, 31.48)	< 0.001
NLR	3.74 (2.775, 5.185)	5.40 (3.27, 12.26)	< 0.001
PLR	165.79 (129.983, 214.580)	188.89 (117.00, 250.00)	0.156
ALT (U/L)	9.00 (6.00, 14.00)	10.00 (7.00, 15.00)	0.136
AST (U/L)	12.00 (9.00, 15.00)	13.00 (10.00, 17.00)	0.069
TP (g/L)	66.60 (62.80, 70.53)	70.30 (64.10, 74.60)	< 0.001
ALB (g/L)	40.20 (37.58, 42.70)	41.00 (37.50, 43.80)	0.329
BUN (mmol/L)	23.54 (18.07, 29.03)	20.12 (14.87, 26.48)	0.003
Scr (umol/L)	787.15 (650.45, 1,010.25)	746.00 (587.00, 905.00)	0.071
CysC mg/L)	5.29 (4.58, 5.91)	4.76 (3.78, 5.38)	< 0.001
UA (umol/L)	368.00 (296.50, 431.75)	365.00 (325.70, 409.00)	0.817
TG (mmol/L)	1.29 (0.91, 2.00)	1.53 (1.14, 2.49)	< 0.001
TC (mmol/L)	3.67 (3.07, 4.25)	3.55 (3.31, 4.04)	0.933
LDL (mmol/L)	1.77 (1.51, 1.89)	1.31 (1.21, 1.44)	< 0.001
HDL (mmol/L)	1.78 (1.25, 2.38)	1.09 (0.86, 1.47)	< 0.001
K (mmol/L)	4.80 (4.26, 5.37)	4.77 (4.24, 5.25)	0.971
NA (mmol/L)	137.60 (135.40, 140.00)	136.70 (134.90, 138.30)	0.053
Ca (mmol/L)	2.155 (2.01, 2.27)	2.180 (2.00, 2.43)	0.288
P (mmol/L)	1.775 (1.41, 2.13)	1.650 (1.32, 2.16)	0.503
Calcium–phosphorus product (mg/dL)	46.90 (36.45, 57.742)	44.02 (33.97, 63.88)	0.498
eGFR (mL/min)	5.60 (4.42, 7.36)	6.10 (4.84, 8.33)	0.095
Hemodialysis vascular access (%)			0.302
Arteriovenous fistula	256 (80.00)	62 (84.93)	
Artificial blood vessel	9 (2.81)	0 (0.00)	
Central venous catheter	55 (17.19)	11 (15.07)	
Total anticoagulant (IU)	4,500.00 (4,000.00, 5,000.00)	4,500.00 (4,000.00, 5,000.00)	0.421
Blood flow rate (ml/min)	240.00 (220.00, 250.00)	250.00 (230.00, 260.00)	< 0.001
Pre-hemodialysis SBP (mmHg)	142.00 (130.00, 155.00)	160.00 (148.00, 173.00)	< 0.001
Pre-hemodialysis DBP (mmHg)	80.00 (75.00, 88.00)	82.00 (77.00, 89.00)	0.237
Post-hemodialysis SBP (mmHg)	139.52 ± 18.03	151.41 ± 20.82	< 0.001
Post-hemodialysis DBP (mmHg)	80.29 ± 8.72	78.44 ± 9.51	0.108

WBCs, white blood cells; PLTs, platelets; HGB, hemoglobin; Ne, neutrophil; Ly, lymphocyte; HCT, hematocrit; CRP, C-reactive protein; NLR, neutrophil-to-lymphocyte ratio; PLR, platelet-to-lymphocyte ratio; ALT, alanine aminotransferase; AST, aspartate aminotransferase; TP, serum total protein; ALB, serum albumin; BUN, blood urea nitrogen; Scr, serum creatinine; CysC, cystatin C; eGFR, estimated glomerular filtration rate; UA, uric acid; TG, triglyceride; TC, total cholesterol; LDL, low-density lipoprotein; HDL, high-density lipoprotein; K, blood potassium; Na, blood sodium; Ca, blood calcium; P, blood phosphorus.

### 3.2. Comparison of the predictive performance of all models

**Figure 2:**
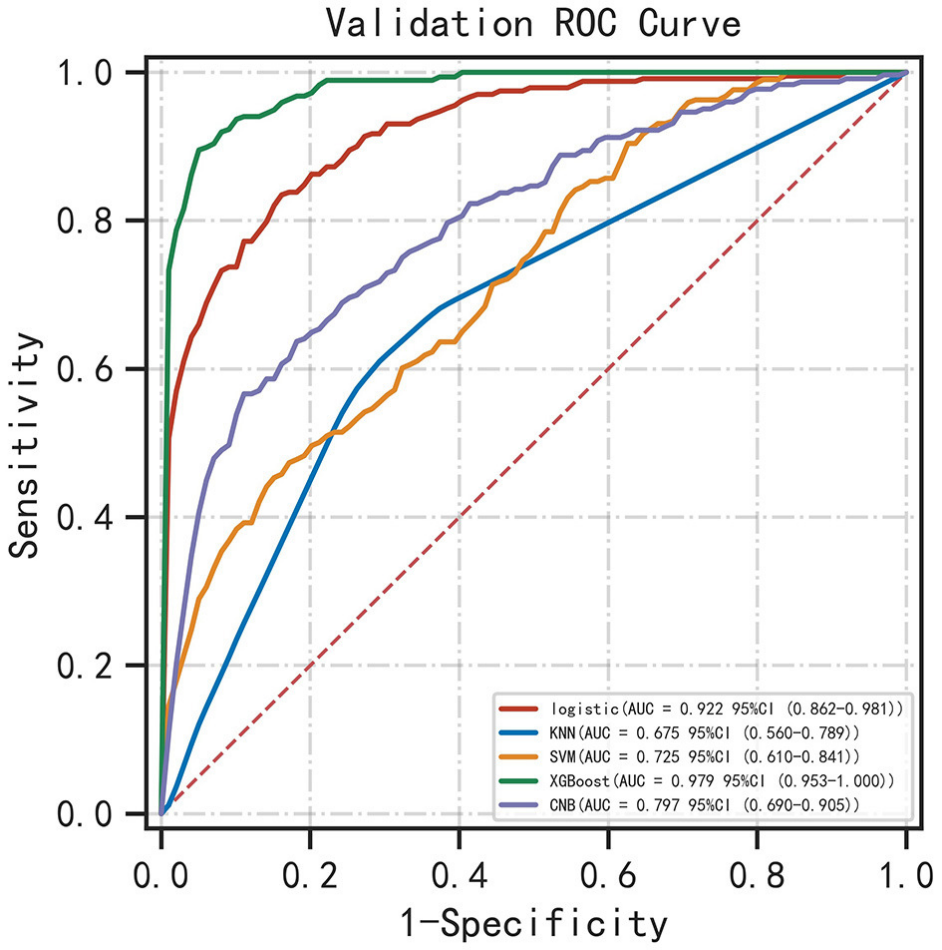
ROC curve demonstrating the performance of ML models in predicting ICH in patients undergoing MHD.

Five ML algorithms were used to construct predictive models. The training set was used to create and train the models. All ML models were tested in the test set, and their accuracy, precision, sensitivity, specificity, and F1 score were compared. The XGBoost model had the highest accuracy, precision, sensitivity, specificity, and F1 score (0.939, 0.949, 0.932, 0.952, and 0.938, respectively) (Table 2). Figure 2 shows a ROC curve demonstrating the predictive performance of all models. The XGBoost model (AUC = 0.979; 95% CI, 0.953–1.000) demonstrated optimal performance in the validation set. Therefore, the XGBoost model can be considered an ideal model for predicting the risk of ICH in patients undergoing MHD.

**Table 2:** Comparison of the predictive performance of five machine learning algorithms in the validation set.

**Different algorithms**	**Accuracy (95%CI)**	**Precision (95%CI)**	**Sensitivity (95%CI)**	**Specificity (95%CI)**	**F1–score (95%CI)**
SVM	0.631 (0.546–0.715)	0.309 (0.272–0.345)	0.753 (0.603–0.902)	0.645 (0.485–0.806)	0.422 (0.377–0.466)
CNB	0.784 (0.741–0.827)	0.457 (0.399–0.514)	0.733 (0.652–0.815)	0.786 (0.716–0.857)	0.556 (0.502–0.610)
KNN	0.785 (0.766–0.803)	0.342 (0.248–0.437)	0.652 (0.599–0.706)	0.695 (0.657–0.733)	0.427 (0.336–0.518)
LR	0.835 (0.813–0.857)	0.539 (0.509–0.568)	0.861 (0.807–0.916)	0.853 (0.799–0.907)	0.659 (0.637–0.682)
XGboost	0.939 (0.926–0.952)	0.949 (0.910–0.988)	0.932 (0.913–0.951)	0.952 (0.930–0.973)	0.938 (0.921–0.956)

SVM, support vector machine; CNB, complement naive Bayes; KNN, k nearest neighbors; LR, logistic regression; XGBoost, extreme gradient boost.

### 3.3. Explainable analysis of overall features

**Figure 3:**
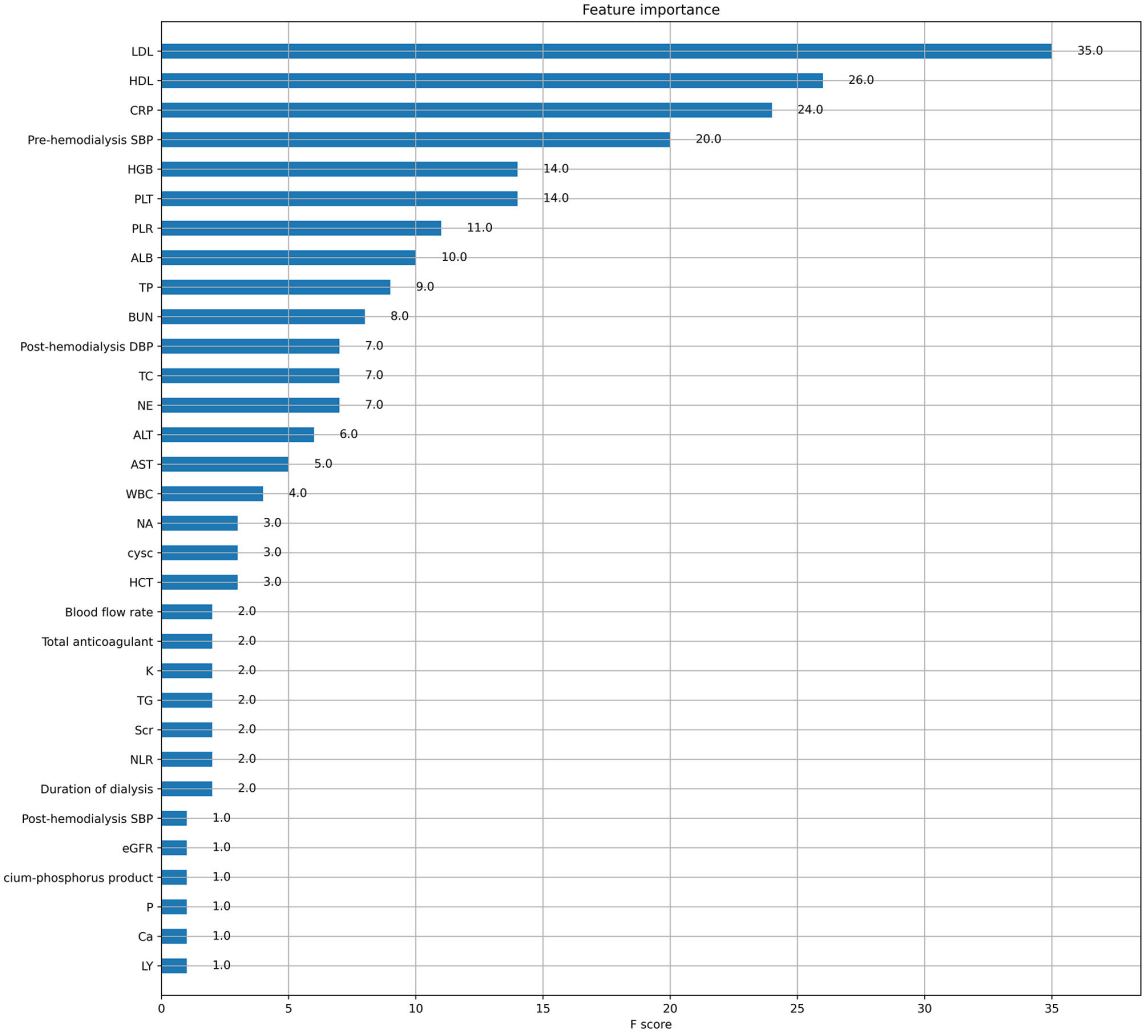
Characteristic ranking of important variables in the model.

XGboost was used to rank the importance of features. Figure 3 shows the ranking of the most important variables in the model. The top five variables were LDL, HDL, CRP, pre-dialysis SBP, and HGB. The interpretation of the impact of these features is roughly consistent with that reported in previous studies and clinician perception.

**Figure 4:**
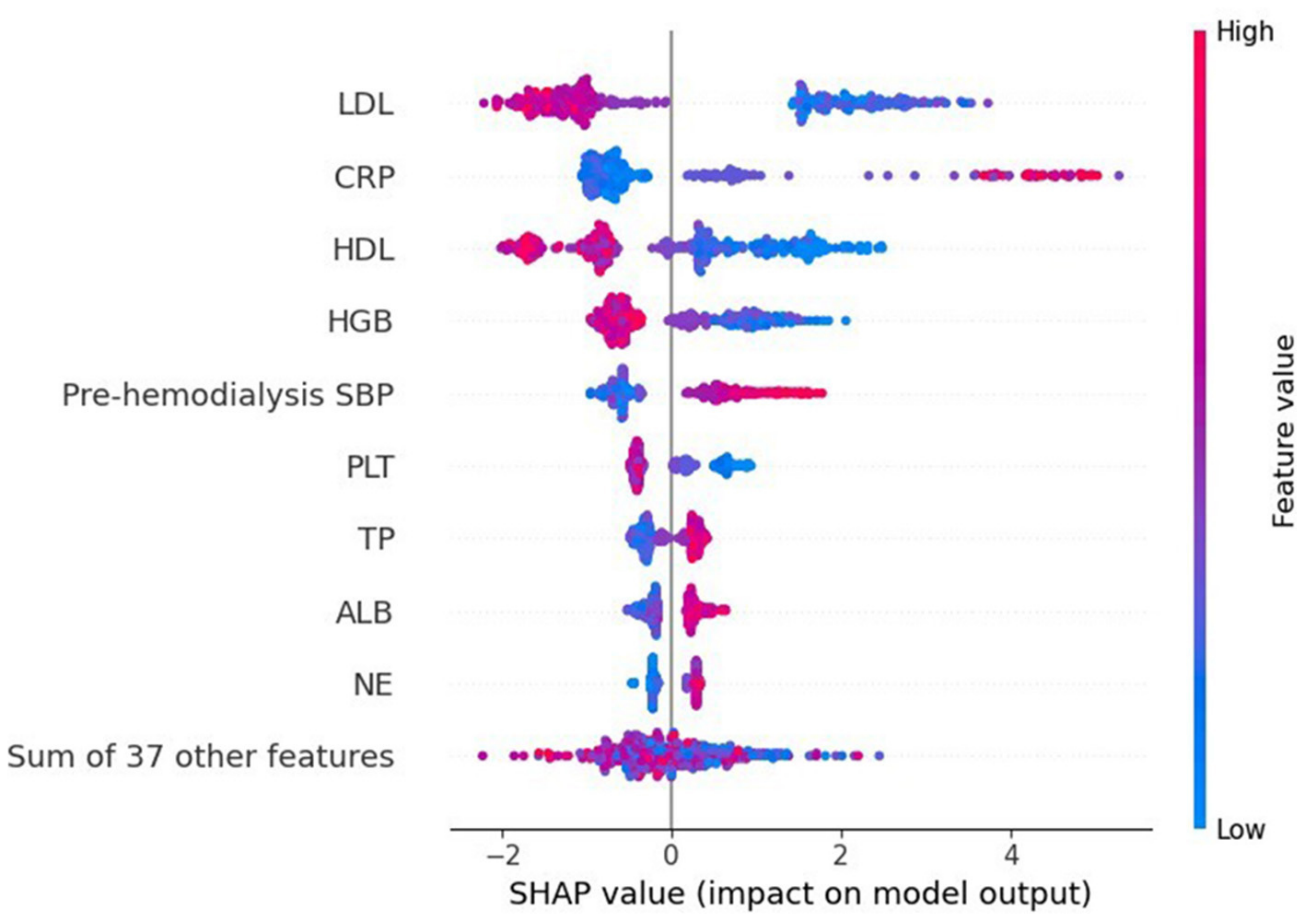
SHAP summary plot of the XGBoost model demonstrates the relationship between each feature in the optimal model (XGboost) and SHAP values. The higher the SHAP value of each feature, the higher the risk of ICH in patients undergoing MHD.

Figure 4 shows a characteristic density scatter plot, which demonstrates the effects of the main features in the dataset on the predictive performance of the model. The abscissa represents the SHAP value, which represents the contribution of a feature in the model to the overall output. SHAP values < 0, equal to 0, and >0 represent negative, no, and positive contributions, respectively. The left ordinate represents the features sorted by importance. The color of the right ordinate, from blue to red, represents the feature values from low to high. Lower LDL levels, higher CRP levels, lower HDL levels, lower HGB levels, and higher pre-dialysis SBP have higher SHAP values, indicating a higher likelihood of developing ICH.

### 3.4. Explainable analysis of individual features

**Figure 5:**
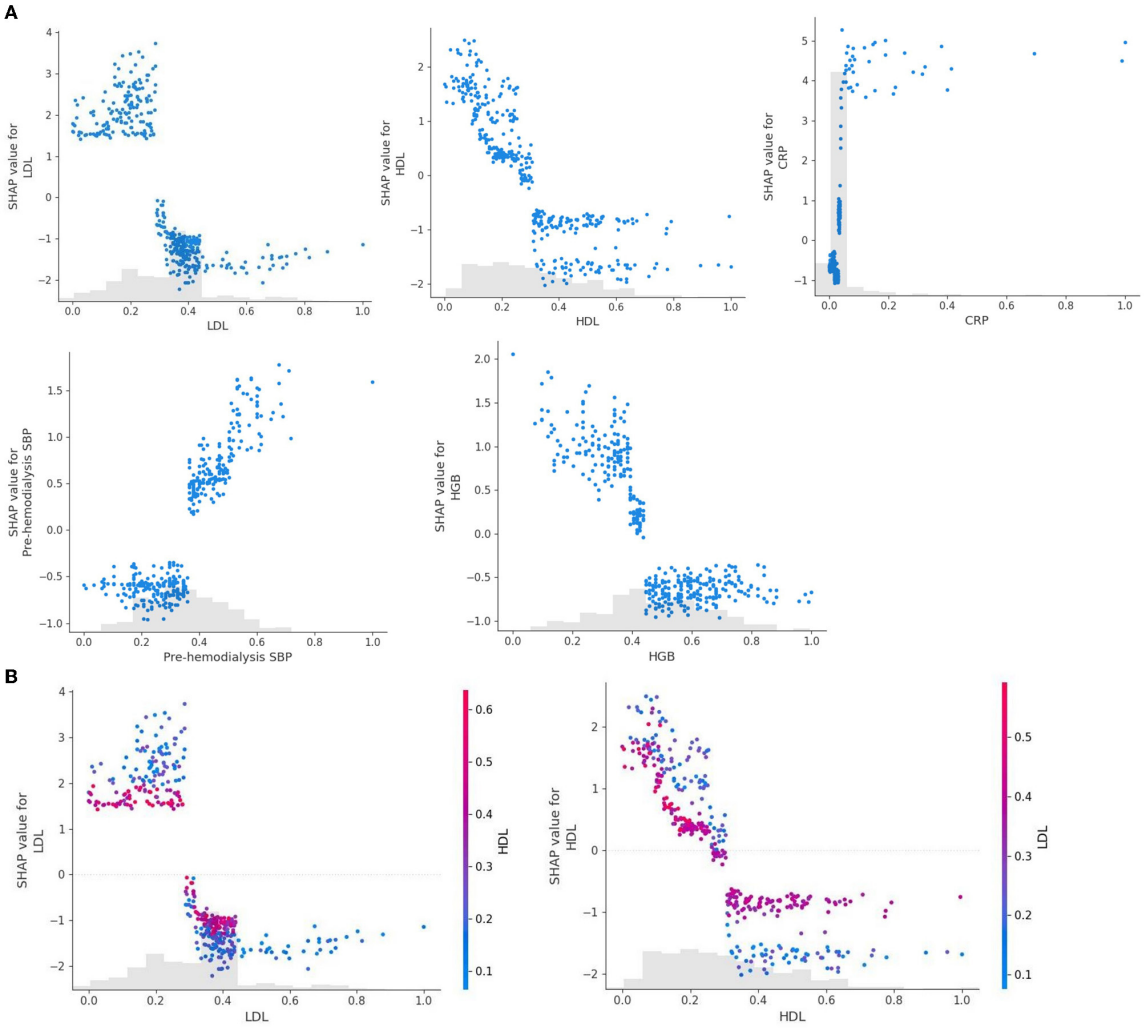
SHAP dependence plot of main indicators. **(A)** The SHAP dependence plot demonstrates the effects of a single feature on the final output of the XGboost model. **(B)** The SHAP dependence plot selects LDL as a feature to determine the effects of HDL.

As shown in Figure 5, the SHAP dependence plot demonstrates the effects of a single feature on the final output of the XGboost model and can be used to select the most significant features of the model. CRP levels and pre-dialysis SBP were positively correlated with SHAP values, that is, the larger the values, the higher the risk of bleeding. However, the levels of LDL, HDL, and HGB were negatively correlated with SHAP values, indicating that the smaller the values, the higher the risk of bleeding (Figure 5A). We selected LDL as a feature to determine the effects of HDL. The red and blue dots represent high and low HDL levels, respectively. After the data were normalized, it was found that when LDL was less than the critical value of 0.3, regardless of HDL levels, the SHAP value of LDL was always greater than zero. In addition, when HDL was greater than the critical value, the SHAP value of HDL was always less than zero (Figure 5B). The cutoff level of LDL is 1.572 mmol/L in actual clinical practice. If this threshold is exceeded, the possibility of ICH decreases. However, if this threshold is not exceeded, the possibility of ICH increases. In addition, the values of all main features are distributed differently in different ranges and vary greatly in some regions. It remains unclear whether these conditions have some specific significance, which may have important implications for clinical outcomes. The feature dependence plot provides information within a given range, showing the trend of possible results. However, it is noteworthy that the plot suggests correlation and not causality. Therefore, it is necessary to integrate the results with clinical experience and specific conditions to determine whether they can be used to develop adjunctive intervention strategies.

**Figure 6:**
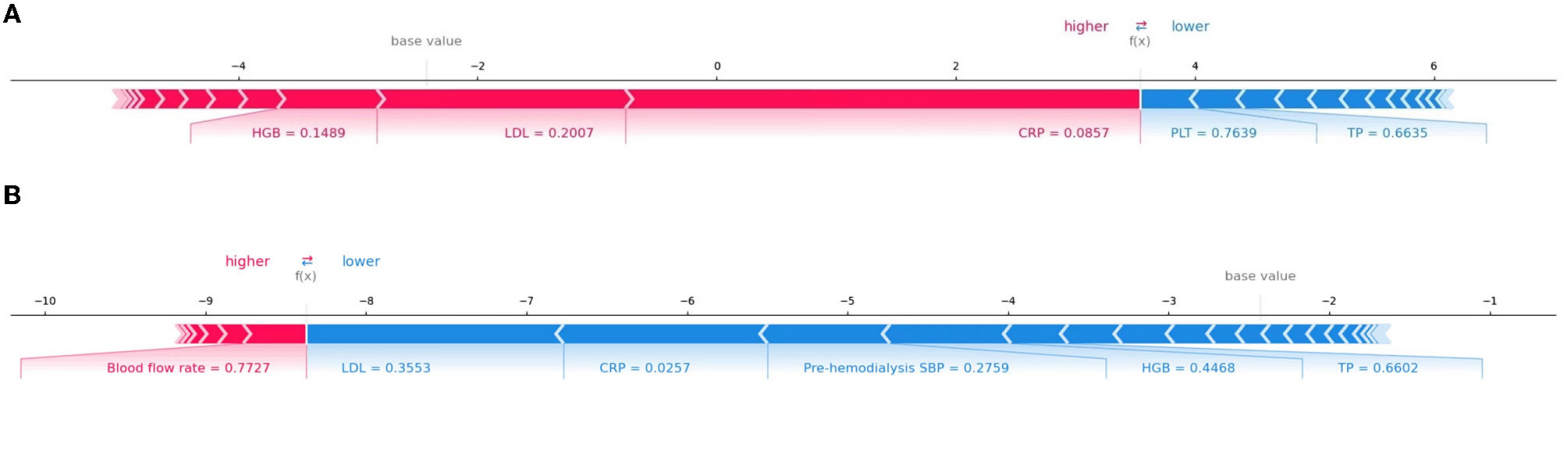
Interpretation of the SHAP model for the prediction of two cases. The red stripe feature is conducive to the prediction of a cerebral hemorrhage in patients undergoing dialysis, whereas the blue stripe feature is conducive to the prediction of no cerebral hemorrhage. **(A)** The contribution of different features to prediction in a patient correctly predicted to have ICH. **(B)** The contribution of different features to prediction in a patient correctly predicted to have no ICH.

In addition, SHAP can be used to analyze the influencing factors of a cerebral hemorrhage in each patient. Figure 6 shows the interpretation of the XGBoost model for the prediction of two cases. Specifically, the arrows show the effects of each factor on prediction. Features that increase the risk of developing ICH are shown in red, and those that reduce the risk are shown in blue. The stripe length of each feature indicates the importance of the feature when making predictions. The longer the stripes, the greater the contribution of the feature to the prediction. After combining the influence of all factors, the corresponding prediction score of each factor was calculated. Figure 6A demonstrates the contribution of different features to prediction in a patient correctly predicted to have ICH. CRP, LDL, and HGB had the largest contribution (red), indicating that they were the main causes of cerebral hemorrhage in the patient. The second patient was accurately predicted to have no ICH (Figure 6B), with LDL, CRP, and pre-dialysis SBP identified as protective factors. Although there were some risk factors, the patient had no cerebral hemorrhage.

## 4. Discussion

Intracerebral hemorrhage is characterized by a high rate of disability and death, which greatly increases the economic burden on families and society, so it is essential to investigate the factors influencing the complications of ICH events in MHD patients. Many scholars have identified the risk factors of ICH and hematoma expansion in patients undergoing MHD and screened variables, such as serum calcium, serum creatinine, and serum antiplatelet agents, *via* multivariate logistic regression (16, 17). Unlike many previous studies, the present study innovatively used ML algorithms to screen for variables, and to the best of our knowledge, this is the first study to report the development of an ML-based predictive model to evaluate the probability of concurrent ICH events in patients undergoing MHD. In addition, we also applied four mainstream machine learning models, namely, LR, SVM, KNN, and CNB, to compare the predictive performance of the XGBoost algorithm with these machine learning methods.

XGBoost is a lifting algorithm based on tree models. Since its establishment in 2016, it has been used to deal with non-linear relationships and complex interactions between variables owing to its higher prediction accuracy and faster operation speed (14). The XGBoost algorithm has been widely used in the medical field, especially for the prediction of critical illnesses. Po-Yu Tseng et al. used the combination of RF and XGboost to predict the risk of acute kidney injury after cardiac surgery, and the final AUC value was 0.843 (8). Pan et al. used XGBoost to predict the mortality of critically ill patients with COVID-19 admitted to the ICU. The AUC values of the training and validation sets were 0.86 and 0.92, respectively (18). The findings of the present study suggest that XGBoost can effectively improve the prediction of ICH in patients undergoing MHD. In this study, the predictors considered to be related to ICH in actual clinical practice and literature were included; patient information was collected as comprehensively as possible; abnormal indicators of various metabolic disorders were refined; ML algorithms were used to analyze variables; and finally, the ROC-AUC value of the optimal model (XGBoost) was as high as 0.979 (Figure 2), with the highest prediction accuracy and significantly better performance than other mainstream machine learning models.

In addition, in this study, we used SHAP to interpret the results of ML models. Emphasis is placed on features that have the greatest impact on outcome measures, thus helping clinicians to realize the rationale behind predicted outcomes early enough to initiate prompt intervention. The results showed that changes in LDL, HDL, CRP, SBP, and HGB levels were the main predictors of ICH in patients undergoing MHD, which was consistent with clinical studies.

Lipid is an indispensable neutral fat in the human body. To date, numerous studies have investigated the relationship between lipid metabolism and ICH. Lipid metabolism disorders in patients undergoing long-term hemodialysis are closely related to the occurrence of a cerebral hemorrhage (19, 20), which is consistent with the results of this study. The Genetic and Environmental Risk Factors for Hemorrhagic Stroke (GERFHS) reported a 33% reduction in the risk of a cerebral hemorrhage in patients with higher cholesterol levels, and a retrospective study (21) reported a significantly increased risk of hemorrhagic stroke in patients with lower HDL levels. The mechanism may be explored because lower LDL-C levels are closely associated with an increased number of cerebral microbleeds (CMBs) (22). Lobar CMBs are mainly associated with cerebral amyloid angiopathy (CAA) (23). The ε 4 allele variation of apolipoprotein E (APOE) is a known genetic risk factor for CAA. Genetic studies have shown a higher rate of reduction in LDL-C concentrations with the APOE ε 4 genotype vector (24). Recent studies have also shown that higher LDL-C genetic risk scores are associated with a higher prevalence of multiple lobar microbleeds (25). CMBs are independent risk factors for ICH and strong predictors of future cerebral hemorrhage (26). In addition, cholesterol is related to physiological processes such as vascular wall construction. Extremely low cholesterol levels may destroy the integrity of intracranial vascular endothelial cells, aggravate vascular endothelial damage, and increase the risk of cerebral hemorrhage (27). HDL is considered a protective factor for atherosclerosis (28), and low HDL levels can aggravate the progression of atherosclerosis, thus increasing the risk of a cerebral hemorrhage.

CRP is an important part of the immune system and one of the signs of acute inflammation (29). In this study, CRP levels were significantly different between the ICH and non-ICH groups, and CRP was highly correlated with ICH, which is consistent with the findings of previous studies (30–32). Patients undergoing MHD often have comorbid inflammation, which may lead to endothelial damage and atherosclerosis (33, 34), thereby increasing the morbidity and mortality of cerebrovascular diseases (35). Genetic studies have shown that the significantly reduced expression of haplotype H5 in the CRP genotype is closely associated with hemorrhagic stroke (36). CRP induces endothelial dysfunction by directly destroying the blood–brain barrier (BBB) and induces monocytes to release proinflammatory cytokines, leading to increased vascular permeability and cerebral hemorrhage (37, 38).

According to the model results of this study, the SBP before daily hemodialysis in the cerebral hemorrhage group was higher than that in the control group, which is consistent with the conclusion that hypertension is a risk factor for cerebral hemorrhage in MHD patients as reported in previous studies. Hypertension is a known traditional risk factor for ICH (39). In patients with chronic kidney disease, renal function and excretion are impaired, blood volume is increased, renin–angiotensin–aldosterone system is activated in a feedback manner, and water and sodium retention is aggravated. In this study, the higher SBP before dialysis in patients with ICH may be related to inadequate dialysis. In addition, during hemodialysis, the greater hemodynamic changes and the excretion of antihypertensive drugs will aggravate hypertension, resulting in increased pressure on cerebral arteries. When the pressure on the vascular wall exceeds the pressure, the cerebral vessels rupture and bleed, causing cerebral hemorrhage.

Patients undergoing MHD are predisposed to anemia owing to factors such as reduced erythropoietin synthesis (40). HGB is the main indicator reflecting the anemic status of humans. Recent studies have reported that the HGB level of patients with MHD is negatively correlated with the risk of a cerebral hemorrhage (41–43), which is consistent with the results of this study. The underlying mechanisms may include vasoconstriction (44), platelet aggregation (45, 46), and cytotoxic reaction caused by chronic hypoxia (44), leading to brain dysfunction or damage.

This study has some limitations. First, although this study had a multicenter design, it only includes patients from three hospitals in Xuzhou, China. In future studies, we will include datasets from different regions and hospitals for external testing to improve the generalization ability of the model. Second, the number of patients with and without ICH was not well-balanced, which may have led to impaired prediction. Considering that deep learning has been widely used in the medical community in recent years, we will use deep learning models to incorporate a wider range of data in future studies. Overall, compared with traditional models, the prediction model developed in this study contains more information and has better predictive accuracy. In addition, the visualization of results based on SHAP can, to a great extent, alleviate the “black box” problem.

## 5. Conclusion

A predictive ML model was developed based on XGBoost, and SHAP was used to explain the clinical significance of each risk factor in predicting the occurrence of ICH in patients undergoing MHD. ICH events in patients undergoing MHD are associated with serum LDL, HDL, CRP, HGB, and pre-hemodialysis SBP levels. The combination of the XGBoost algorithm and SHAP can provide a clear explanation for risk prediction, which has great application value in future clinical research. This combination can help clinicians to implement early clinical interventions, provide more comprehensive information for the long-term management of patients undergoing MHD, and prevent and reduce the risk of ICH.

## Data availability statement

The raw data supporting the conclusions of this article will be made available by the authors, without undue reservation.

## Ethics statement

The studies involving human participants were reviewed and approved by the Ethics Committee of the Affiliated Hospital of Xuzhou Medical University. Written informed consent from the patients/participants or patients/participants' legal guardian/next of kin was not required to participate in this study in accordance with the national legislation and the institutional requirements.

## Author contributions

FL and AC conceptualized the study, outlined the study design, collected data, analyzed and interpreted results, and wrote the manuscript. ZL and QP preprocessed input data, built machine learning models, analyzed data, and wrote the manuscript. LG collected data and preprocessed input data. YF, JF, and PW helped to adjust the ideas of the manuscript, suggested changes, and revised the manuscript. All authors agreed to take responsibility for their contributions and read and approved the final manuscript.
